# Communication patterns in families affected by parental cancer from the healthy parents’ perspective—process evaluation of the complex intervention Family-SCOUT

**DOI:** 10.1007/s00520-024-08705-x

**Published:** 2024-07-10

**Authors:** L. Heier, J. Weiß, C. Heuser, H. Nakata, E. Brock-Midding, R. Horbach-Bremen, T. H. Brümmendorf, M. Brüne, M. Dohmen, B. Drueke, F. Geiser, S. Holsteg, A. Icks, A. Karger, J. Panse, A. Petermann-Meyer, A. Viehmann, N. Ernstmann

**Affiliations:** 1 Department of Psychosomatic Medicine and Psychotherapy, Center for Health Communication and Health Services Research, University Hospital Bonn, University Bonn, Bonn, Germany; 2 Center for Integrated Oncology, Aachen Bonn Cologne Düsseldorf (CIO-ABCD), Aachen, Germany; 3 Department of Clinical Pharmacy and Toxicology, Maastricht University Medical Center, Maastricht, The Netherlands; 4 Department of Clinical Pharmacy, Cardiovascular Research Institute Maastricht, CARIM, Maastricht University, Maastricht, The Netherlands; 5 Institute of Medical Sociology, Health Services Research, and Rehabilitation Science, Chair of Health Services Research, Faculty of Medicine and University Hospital Cologne, University of Cologne, Cologne, Germany; 6 Department of Hematology, Oncology, Hemostaseology and Stem Cell Transplantation, Faculty of Medicine, RWTH Aachen University, Aachen, Germany; 7 Center for Health and Society, Faculty of Medicine, Heinrich-Heine-University Düsseldorf, Düsseldorf, Germany; 8 Department of Psychosomatic Medicine and Psychotherapy, University Hospital Bonn, University Bonn, Bonn, Germany; 9 Clinical Institute of Psychosomatic Medicine and Psychotherapy, Medical Faculty and University Hospital Düsseldorf, Heinrich-Heine-University Düsseldorf, Düsseldorf, Germany; 10 Institute for Health Services Research and Health Economics, German Diabetes Center, Leibniz Center for Diabetes Research at Heinrich-Heine-University Düsseldorf, Düsseldorf, Germany; 11 German Center for Diabetes Research, Partner Düsseldorf, Munich-Neuherberg, Germany; 12 Institute for Medical Psychology and Medical Sociology, Faculty of Medicine and University Hospital, RWTH Aachen University, Aachen, Germany

**Keywords:** Parental cancer, Minor children, Family intervention, Family-SCOUT, Qualitative research, Template analysis, Health services research

## Abstract

**Purpose:**

Within families affected by parental cancer, open communication impacts the well-being of parents and their children; however, limited research exists on communication patterns in these families. This sub-study addresses this through the Family-SCOUT study, a multicenter, prospective, interventional, and non-randomized investigation with intervention (IG) and control group (CG). The purpose of this sub-study was to identify and compare the differences in communication patterns between the IG and CG as part of the process evaluation. The research question was addressed in both groups: What communication patterns do healthy parents perceive within their families?

**Methods:**

Using a qualitative approach, the study involved interviewing healthy parents as surrogates for their families. The interviews were audio-recorded, transcribed, and coded using a template analysis. The resulting data were analyzed at the group level.

**Results:**

Twenty-three interviews were conducted in the IG and 27 interviews in the CG. The analysis of themes centered on communication patterns as seen in the family structure. Both groups exhibited instances of open communication about fears and wishes as well as the use of child-friendly language when discussing cancer. Notable differences were observed: challenges in open communication with children were sorely reported in CG interviews, and “the illness is discussed when necessary” was sorely described in IG interviews.

**Conclusion:**

This study underscores the need to address and encourage open communication within families with parental cancer.

**Supplementary Information:**

The online version contains supplementary material available at 10.1007/s00520-024-08705-x.

## Introduction

The burdens in families affected by parental cancer are high for the sick as well as for the healthy parent, but also for the children: responsibilities, roles, and routines may change, sudden financial pressure, and/or dealing with a potentially life-threatening disease, development of psychosocial stress may occur [1–4]. Although 37,000 parents of minor children are diagnosed with cancer each year in Germany [5], little is known about family functioning among parents with cancer who have minor children [6]. Park and colleagues explored five patterns of family management in parental advanced cancer: equipped and optimistic, equipped and pragmatic, discouraged and struggling, apprehensive and passive, discouraged and conflicted [6]. The identified patterns are shaped by communication within the family, from proactive and open communication about the disease to minimizing discussions about serious consequences. Communication patterns have emerged as a central aspect of family dynamics, playing a pivotal role in how families navigate the complexity of emotions, uncertainties, and coping strategies. The way information is shared, feelings are expressed, and support is sought can either enhance or hinder the family’s collective ability to adapt to the challenges posed by cancer. One of the main factors which is responsible for the overall adjustment of children to parental cancer is the communication with their parents and family [7, 8]; therefore, it is crucial to gain a better understanding of the nuances of communication within these families and to identify opportunities for targeted interventions and support mechanisms to bolster their resilience and well-being during this period.

In Germany, an actively outreaching, family-centered, cross-sectoral intervention “Family-SCOUT” was developed within the Center of Integrated Oncology Aachen together with the regional Caritas association, to address the unmet needs of families. The intervention is based on dedicated multi-professional comprehensive care and case management with a permanent contact person (the so-called family scout) [9, 10]. The intervention was provided through home visits, telephone support, text/email messages, or video calls and included organizational support (e.g., arranging household help or advising on securing finances), communicative support (e.g., offering age-appropriate cancer information for children), and emotional support (e.g., developing effective coping strategies) [10]. Within the Family-SCOUT study, the present evaluation aims to identify and compare differences in communication patterns between the two study arms (intervention group (IG) and control group (CG)) as part of the process evaluation. To achieve this, the following research question was addressed in both groups: What communication patterns do healthy parents (HP) perceive within their families?

## Methods

### Study design

The Family-SCOUT study had a quasi-experimental, non-randomized, unblinded, prospective superiority control group design with two study arms and took place within the Comprehensive Cancer Center, Network Center of Integrated Oncology Aachen-Bonn-Cologne-Düsseldorf (CIO^ABCD^), in three of the four locations—Aachen, Bonn, and Düsseldorf [9, 10]. While quantitative data were collected for a summative assessment, the study involved 50 semi-structured interviews conducted with 32 families, encompassing both the intervention and control groups. The interviews were intended for a process evaluation of the impact of the intervention as well as getting insight into burdens, communication patterns, and resources within the families.

All interviews were carried out by two female members of the evaluation team, both holding advanced degrees, one in Sociology and Social Research and the other in Public Health. These researchers received extensive training in collecting, managing, and analyzing qualitative data. No direct personal contact was made with the participants prior to the interviews.

### Participant selection

Participant recruitment was conducted between January 2019 and December 2020 at four study sites: Bonn, Aachen, Düsseldorf, and Bad Oeynhausen (only and exclusively for the process evaluation). The inclusion criteria for participation in the project were that at least one parent had an ICD-10 C diagnosis, one or both had custody of at least one minor child, and proficient in the German language [9]. The exclusion criterion was the withdrawal of consent [9]. Purposive sampling was employed to ensure a diverse range of interviewees, considering factors such as gender, number of children, perceived burden, and educational background. Due to the changes caused by the pandemic and the (temporary) stop of further data collection, the study team decided to conduct longitudinal interviews, which were not previously planned in the study protocol. All families who were already interviewed were contacted again to invite them for a second interview. The goal was to create a sample that represents the heterogeneity of the study population.

### Data collection

The Family-SCOUT study was introduced, data protection documents were explained, and the interview procedure and open questions were clarified. Interviews with IG families were conducted after the families had been included in the study during or after the completion of the intervention. Interviews with CG families were conducted after inclusion in the study. There was no relationship between interview timing and data analysis and the quantitative measurement points. Interview timing was based on family availability. The interview subjects revolved around three themes: familial circumstances in light of a parent’s illness (burdens and resources), dynamics of communication within the family, and the perceived need for support. All interviews were recorded, transcribed, and pseudonymized. The transcripts were not presented to the participants.

### Data analysis

Template analysis was carried out as a form of thematic analysis [11, 12]. The aim is to create a coding template that is generated by analyzing part of the qualitative data, then applying it to the rest of the data, revising it, and applying it again [11, 12]. The key aspects of this technique are as follows: (1) flexibility of the coding structure and (2) the creation of a priori themes and (3) original template. The methodological steps were as follows: (A) becoming familiar with the data material. (B) Preliminary coding of qualitative data. In this process, evaluators consider all text segments that are considered important. (C) Organizing themes into meaningful clusters and defining how themes relate to each other within these groups. (D) Definition and creation of a preliminary coding template. (E) Apply the preliminary template to the full qualitative data and revise and reapply it. (F) Finalizing the coding template.

## Results

50 interviews were conducted with 32 families. Overall, the interviews lasted a median of 29 min and a mean of 30 min, with a range of 10 to 63 min. 32 initial interviews were conducted, 16 in the CG and 16 in the IG. In addition, 18 longitudinal interviews were conducted, 11 in the CG and seven in the IG. An overview of the distribution can be found as supplementary material.

On average, the participants were 48 years old. 17 women and 15 men were interviewed, of whom 12 had a university entrance certificate, eight had an intermediate secondary school education, six had an entrance certificate for a university of applied science, and two had another (undefined) qualification. One participant had a lower secondary school certificate. Three of the interview participants did not provide any information regarding their qualifications. two interviews with grandparents representing the family, one interview with a child (18 years), and 28 interviews with HP. In one interview, the child’s biological mother was not interviewed, but the stepmother as an HP. A detailed overview of the sample descriptions, treatment approach of the sick parent, and year of first cancer diagnosis can be found as supplementary material.

We identified three main themes and 11 subthemes. Table 1 provides an overview of the template with main themes, subthemes, and the distribution in the IG and CG.

**Table 1:** Coding template

Themes	Subthemes	Both groups	CG	IG
1. Open communication	1.1 Open exchange through daily conversations	x		
	1.2 Challenges and difficulties in open communication with children		x	
2. Lack of communication	2.1 No confrontation about the disease in the family	x		
	2.2 No Communication with children	x		
	2.3 Lack of grief communication			x
	2.4 No communication about last phase of life			x
	2.5 Internal role conflicts and excessive demands		x	
3. Closed communication	3.1 Illness is discussed internally		x	
	3.2 Unclear agreements		x	
	3.3 The disease is discussed when necessary			x
	3.4 Lack of cooperation			x

Both groups, CG and IG: x shows whether the themes and subthemes are mentioned.

### Theme 1: Open communication

Open communication covers the subthemes 1.1 open exchange through daily conversations about the disease and 1.2 challenges and difficulties in open communication with children.

Communication patterns within this theme were described as regular conversations about all aspects of the disease and challenges and changes regarding all family members. Conversations focus on positive as well as negative aspects, burdens, and organizational challenges. What is important here is honesty, openness, and trust within the family.

Relatively open and frequent. That just goes beyond everyday things: ‘What can be done, what needs to be done?,’ precisely because my wife can no longer move so is restricted. Up to conversations with undertakers about what a funeral could look like. But also practical questions now: ‘Okay, what do we perhaps have to do, if we have a bit more time now, what do we perhaps have to change in our house to make everyday life easier for my wife in particular, but also for all of us?’ (IG014)

We talk about it normally, without mincing our words. But we’ve always talked openly about all things, whatever the topic is (CG025).

The subtheme 1.2 challenges and difficulties in communication with children was explicitly mentioned by HP in CG. Uncertainties about how to explain the disease in a child-friendly way but also how much should be explained. How can the parents inform and protect their children was one of the leading questions by the HP. The desire to reduce the burden on the children adjusting to the truth was a problem, which come along with open communication with the children of CG.

We are very careful not to use the word cancer or the word chemo or radiotherapy […]. If it can’t be avoided, we like to switch to English so as not to confront him with (CG025).

### Theme 2: Lack of communication

Lack of communication focuses on the subthemes 2.1 no confrontations about the disease in the family, 2.2 no communication with children, 2.3 lack of grief communication, 2.4 no communication about last phase of life, and 2.5 internal role conflicts and excessive demands.

The subthemes 2.1 no confrontations about the disease in the family and 2.2 no communication with children are mentioned from HPs of IG and CG. Both subthemes are focusing on missing communication about the disease and avoidance and blocked conversation within the families and especially with children. HP report several attempts to talk about the changes in their lives and in the family, failed conversations and avoidance of family members to participate in the conversations.

We started to deal with it completely differently. And now there’s quite a bit of speechlessness. We both have our own way of dealing with it. I sometimes find it difficult to accept that my husband, I always assume that he’s pushing it. He just has a slightly different, more optimistic attitude than me. He gets angry when I cry, he forbids me to do that. So there’s no more togetherness (IG022).

My husband, a few weeks before he died, he was in the clinic for chemo. And his whole wrist was quite blue. And then of course the children asked: “Dad, what did you do there?” [...] And I said: “That’s the most normal thing in the world if you explain to them, even if we don’t use the word, if we don’t say chemo or cancer, […] listen, they gave me an IV. That’s why my hand is blue.” And then he told them things like that he had run into the door (CG009#2).

Subthemes 2.3 lack of grief communication and 2.4 no communication about last phase of life was mentioned sorely in IG. Both subthemes are focusing on topics regarding end-of-life care and death of the SP in IG and conversations do not take place in the families. These aspects are associated with a high level of subjectively perceived stress within the family, unspoken feelings and beliefs of family members, and unequal information sharing within the family system.

He said: “What are you thinking about?” I was like: “You want your two children to be looked after.” And: “Yes, but I’m not going to worry about that yet.” And then I said to him: “Well, you know, you’ll be dead one day. And then the story will be over for you. For us, there is an after. […] And we also have to talk about the after, you’ll have to deal with that too. Because then you’re dead, you’re out of the game, great.” (IG022)

Subtheme 2.5 internal role conflicts and excessive demands was a subtheme and was mentioned of HP in CG. In relation to lack of communication within the family, the HP of CG raises the issue of internal role conflicts and the resulting excessive demands of family members. HP describes his or her role as the healthy parent, husband/wife, contact person for the children, and the wider family. The burdens associated with new and old roles and changing daily life because of cancer.

She sees me at that moment not so much as a mother, really as a professional. […] But I’m not the expert for our daughter. I’m the mother. […] On the one hand it’s a great feeling to be needed like that, but it also takes away part of my role as a mother (CG006).

### Theme 3: Closed communication

Closed communication within the family is covering four subthemes: 3.1 illness is discussed internally, 3.2 unclear agreements, 3.3 the disease is discussed when necessary, and 3.4 lack of cooperation.

Subthemes 3.1 illness is discussed internally and 3.2 unclear agreements are discussed by HP of CG. The disease and how it affects the entire family system is discussed internally, and there are hardly any discussions with external parties, e.g., the social network or healthcare provider. The disease is perceived as something very personal, which only concerns the core family. This form of closed communication is seen as protection, as a way of gaining control over something uncontrollable.

We were more likely to leave it within the family. Crap. Rather left it within the family (CG027)

Subthemes 3.3 the disease is discussed when necessary and 3.4 lack of cooperation were, on the other hand, mentioned by HP of IG. It was about the lack of togetherness as a family, and that the disease was only talked about when necessary. Both aspects are related to a closed communication, which was considered not only a burden but also a factor in managing everyday life for the family as a system. The disease was not ignored completely; however, discussions about this were reduced to a minimum.

The main topic is not always the subject of cancer and death. That comes up from time to time, or that he’s missing. But we don’t make cancer the main focus here in the family. The illness just sucks. And, yes, life goes on and we now have children’s communion (IG005).

## Discussion

Aiming to analyze communication patterns of families affected by parental cancer with the help of template analysis, 50 interviews with 36 healthy parents were conducted. We identified three communication patterns: open communication, lack of communication, and closed communication. The patterns are characterized by the degree of openness and honesty within the communication, relevance in everyday life, and the willingness of individual family members to deal with the disease through communication within the family system. The use of open communication regarding all aspects of cancer was reported as a regular occurrence. This included discussing both positive and negative aspects of the disease, as well as daily life circumstances and topics unrelated to the cancer of a parent. It was identified as a valuable resource for the family. In the CG, challenging conversations and insecurities surrounding child-friendly communication were highlighted as an important aspect of their communication patterns.

The main driver for the decision to have open or closed communication about the illness was described as the desire to be a good parent [13]. The main aim of the ill parents is to protect their family [1]. Communication is one of the key elements to help children deal with the illness and is described as the main concern of mothers when they are diagnosed with cancer [1, 14]. A study on the experiences of parents when the mother has cancer was published and described open communication as an important resource for the whole family [15]. However, challenges regarding open communication affect individual family members as well as the global family system. Whether the result that uncertainties regarding open communication with children were primarily mentioned by HP in the CG is related to the intervention must be examined in further studies. This result indicates that communication with children may have been discussed more openly in IG. However, we cannot conclude on the basis of this analysis whether this is an effect of the intervention.

Communication about topics that are not related to the disease in the first place, keeping routines, and establishing new ones can be a resource for the family system, which may be difficult to keep up during the cancer journey [4, 16, 17]. In this study, HP describes closed communication as the possibility of having conversations about cancer of the sick parent, but is not the main focus of everyday communication within the family system. Closed communication was described as a protective shield close to the border of the second pattern of a lack of communication.

HP describes lack of communication as avoiding communication about the disease, the current situation within the family system, and grief and/or conversations about the last phase of the SP. Missing communication with the children and family was mentioned by the HP of both study arms. Conflicts about financial security, fear, and concerns are not addressed or blocked by a parent. Children refuse to talk about their own feelings or illnesses regarding the SP or parents refuse to talk to their children about the disease. Zhang et al. reported similar findings regarding avoided communication between mothers with breast cancer and their children [18]. Yu et al. report in their systematic review that most parents with cancer finally discuss their illness with their children, even though they also report a wish to learn how to communicate the disease first [19].

### Limitations

This study encountered a methodological limitation in utilizing a healthy parent as the primary interviewee, who served as a proxy for the family system. From a methodological standpoint, our interviews did not provide a holistic family perspective. The difficulty faced by healthy parents in conveying the family’s viewpoint vicariously was highlighted. Future studies should focus on the family perspective of all family members’ experiences. On the other hand, the exclusive perspective of a healthy parent is a resource that has so far rarely been used to describe the family’s experience when a parent has cancer.

It must also be discussed what effect non-randomization has on the results. Families in the IG may have explicitly asked for support in terms of open communication within the family, although organizational aspects were more of a priority at the beginning of the intervention. This potential selection bias must be taken into account when interpreting the results.

The objective was to identify everyday experiences, burdens, resources, and communication patterns within the family. However, there was no time reference for the course of the intervention, illness, or therapy, and we conducted longitudinal interviews with healthy parents but did not analyze the interviews over time. Further studies are needed to investigate how communication patterns change during the course of an illness and to draw conclusions about the pattern of communication and degree of perceived burden within the family.

## Conclusion

We have identified three distinct communication patterns: open communication, lack of communication, and close communication. Notable differences were identified as challenges in open communication with children (CG) and how intensely the illness is discussed, when necessary (IG). In order to support families during their cancer journey, family-centered interventions like Family-SCOUT manual as well as parent-centered interventions are existing [20–22]. Beyond that, healthcare providers must aware of this potential flaw in communication and prioritize offering guidance to promote more open communication.

### Supplementary Information

Below is the link to the electronic supplementary material.

Supplementary file1 (PDF 187 KB)

## Data Availability

The data supporting the conclusions of this article will be made available by the authors on reasonable request, without undue reservation.
