# Supplementary material for: Communication patterns in families affected by parental cancer from the healthy parents’ perspective—process evaluation of the complex intervention Family-SCOUT
Source: Support Care Cancer. 2024 Jul 10;32(8):500. doi: 10.1007/s00520-024-08705-x (PMC11236872; doi:10.1007/s00520-024-08705-x)
Supplement: Supplementary file 1 — Supplementary file1 (PDF 187 KB) [file 520_2024_8705_MOESM1_ESM.pdf]

Communication patterns in families affected by parental cancer from the healthy parents' perspective – process evaluation of the complex intervention Family-SCOUT

Journal: Supportive Care in Cancer

Heier, L., Weiß, J., Heuser, C., Nakata, H., Brock-Midding, E., Horbach-Bremen, R., Brümmendorf, T.H., Brüne, M., Dohmen, M., Drueke, B., Geiser, F., Holsteg, S., Icks, A., Karger, A., Panse, J., Petermann-Meyer, A., Viehmann, A., Ernstmann, N.

Corresponding author:

lina.heier@mumc.nl

Center for Health Communication and Health Services Research, Department of Psychosomatic Medicine and Psychotherapy, University Hospital Bonn, University Bonn, Bonn, Germany

Center for Integrated Oncology, Aachen Bonn Cologne Duesseldorf (CIO-ABCD), Germany

Table 1: Overview of the interviews

	**N**	**Length (median)**	**First interview (n)**	**Length (first interview) (median)**	**Second interview (n)**	**Length (second interview) (median)**
Study	50	29 minutes	32	29 minutes	18	27 minutes
Intervention Group (IG)	23	32 minutes	16	32 minutes	7	29 minutes
Control Group (CG)	27	26 minutes	16	23 minutes	11	25 minutes

Table 2: Sociodemographic factors of the interview partners in **IG**

**ID**	**Age (between)**	**Gender**	**Education**	**Net household income**	**Age first child (years)**	**Age second child (years)**	**Age third child (years)**	**Treatment approach of cancer parent**	**Year of first diagnosis of cancer parent**
002	50 – 59	Male	Intermediate secondary school education	Missing	11 – 15	None	None	Palliative	2012
003	60 – 69	Male	Lower secondary school qualification	1.500 € - 2.000 €	11 - 15	11 - 15	None	Missing	2018
004	30 - 39	Female	Entrance certificate for a university of	3.000 € - 3.500 €	≤ 5	None	None	Curative	2018

			applied science						
005	50 – 59	Female	Intermediate secondary school education	2.000 € - 2.500 €	6 – 10	None	None	Palliative	2017
014	50 – 59	Male	University entrance certificate	4.000 € - 4.500 €	11 - 15	None	None	Palliative	2019
015	50 – 59	Female	Intermediate secondary school education	2.500 € - 3.000 €	16 - 20	None	None	Palliative	2019
019	50 – 59	Female	Entrance certificate for a university of applied science	4.000 € - 4.500 €	6 – 10	16 - 20	None	Palliative	2016
022	40 – 49	Female	Intermediate secondary school education	2.500 € - 3.000 €	11 - 15	16 - 20	None	Curative	2018
023	30 – 39	Female	Intermediate secondary school education	1.500 - 2.000 €	≤ 5	None	None	Missing	2019
028	30 – 39	Female	Missing	2.000 € - 2.500 €	≤ 5	None	None	Curative	2020
029	40 – 49	Male	Intermediate secondary school education	Missing	≤ 5	None	None	Curative	2016
030	40 – 49	Male	Intermediate secondary school education	3.500 € - 4.000 €	11 - 15	16 - 20	None	Curative	2020
031	50 – 59	Female	Entrance certificate for a university of applied science	3.000 € - 3.500 €	11 - 15	11 - 15	16 – 20	Curative	2020
032	<20	Male	Missing	Missing	Missing	Missing	Missing	Curative	2020
033	40 – 49	Female	University entrance certificate	3.000 € - 3.500 €	6 – 10	None	None	Missing	2020
034	40 - 49	Male	University entrance certificate	4.000 € - 4.500 €	11 - 15	None	None	Curative	2020

Table 3: Sociodemographic factors of the interview partners in **CG**

**ID**	**Age**	**Gender**	**Education**	**Net household income**	**Age first child (years)**	**Age second child (years)**	**Age third child (years)**	**Treatment approach of cancer parent**	**Year of first diagnosis of cancer parent**
001	40 - 49	Female	University entrance certificate	> 8000 €	6 – 10	6 – 10	None	Palliative	2010
006	60 - 69	Female	University entrance certificate	2500 € - 3000 €	Missing	Missing	Missing	Missing	Missing
007	40 - 49	Male	University entrance certificate	3.000 € - 3.500 €	≤ 5	≤ 5	None	Curative	2018
008	30 - 39	Female	University entrance certificate	3.000 € - 3.500	≤ 5	None	None	Curative	2019
009	30 - 39	Female	Missing	2000 € - 2500 €	6 – 10	16-20	None	Palliative	2016
010	30 - 39	Female	Missing	4000 € - 4500 €	≤ 5	None	None	Curative	2004
011	30 - 39	Male	University entrance certificate	3500 € - 4000 €	6 – 10	None	None	Missing	2019
012	40 - 49	Male	University entrance certificate	6000 € - 8000 €	6 – 10	6 – 10	≤ 5	Curative	2018
013	40 - 49	Male	Intermediate secondary school education	Missing	6 – 10	6 – 10	None	Palliative	2014
016	30 - 39	Male	Intermediate secondary school education	3000 € - 3500 €	≤ 5	None	None	Curative	2019
017	40 - 49	Female	Entrance certificate for a university of applied science	2500 € - 3000 €	6 – 10	None	None	Curative	2018
021	60 - 69	Female	University entrance certificate	3500 € - 4000 €	16 – 20	11 - 15	None	Palliative	2012
024	40 - 49	Female	University entrance certificate	Missing	11 – 15	11 - 15	6 – 10	Missing	Missing
025	40 - 49	Male	Entrance certificate for a university	5000 € - 6000 €	≤ 5	≤ 5	none	Curative	2020

			of applied science						
026	60 - 69	Female	Entrance certificate for a university of applied science	Missing	≤ 5	None	None	Missing	Missing
027	40 - 49	Male	University entrance certificate	Missing	6 – 10	6 – 10	≤ 5	Missing	Missing
